# Do Not Throw Away Your Shot: Pilot Study in Improving Medical School Curricula Through Focused Vaccine Education

**DOI:** 10.1016/j.focus.2023.100178

**Published:** 2023-12-23

**Authors:** Jorna Sojati, Anjana Murali, Glenn Rapsinski, John V. Williams

**Affiliations:** 1 Department of Pediatrics, School of Medicine, University of Pittsburgh, Pittsburgh, Pennsylvania; 2 Department of Microbiology and Immunology, School of Medicine, University of Pittsburgh, Pittsburgh, Pennsylvania; 3 Department of Medicine, School of Medicine, University of Pittsburgh, Pittsburgh, Pennsylvania; 4 Department of Microbiology & Molecular Genetics, School of Medicine, University of Pittsburgh, Pittsburgh, Pennsylvania; 5 Institute for Infection, Immunity, and Inflammation in Children, UPMC Children's Hospital of Pittsburgh, Pittsburgh, Pennsylvania

**Keywords:** Medical education, vaccine education, education research, vaccines

## Abstract

**Introduction:**

With widely prevalent vaccine hesitancy and medical mistrust, future healthcare providers require knowledge of vaccines and comfort in discussing vaccines with patients. However, many U.S. medical schools do not offer formal or elective curricula focused on vaccine education.

**Methods:**

We sought to identify the need for such a curriculum and student gaps in knowledge through a pilot study surveying first- and second-year medical students at a large Pennsylvania medical school to assess knowledge about vaccine initiatives/policies, comfort in discussing vaccines, attitudes toward curricular changes, and effects of COVID-19 on willingness for vaccine education.

**Results:**

Many participants (>40%) reported insufficient knowledge of vaccine policy, vaccine development and testing, and community vaccine initiatives. The majority (>50%) indicated discomfort in discussing vaccines in a clinical setting. Importantly, 79% of participants reported insufficient coverage of vaccine topics in the current curriculum. A total of 54% decisively wanted formal/mandatory education versus 72% who decisively wanted elective education. Desiring formal education was associated with less comfort in discussing vaccines overall and with vaccine-hesitant people in clinical settings. Reassuringly, 74% of participants noted that the COVID-19 pandemic made them want to learn more about vaccines. Guided by these findings, we conducted a pilot elective teaching about vaccines through case studies, interactive discussions, and role-playing scenarios. Pre- and postcourse surveys showed improvement in student knowledge in all vaccine-related topics assessed.

**Conclusions:**

These data can inform the development of formal and elective curricula that effectively prepare medical students for patients’ vaccine questions.

## HIGHLIGHTS

• Medical students were surveyed after completing curricular coverage of vaccines.
• A total of 79% of students report insufficient coverage of vaccine topics in the current curriculum.
• A total of 54% want more formal/mandatory vaccine education, and 72% want elective education.
• Wanting formal education was associated with discomfort in discussing vaccines clinically.
• Pilot elective teaching informed by survey data improved student knowledge of vaccine topics.

## INTRODUCTION

The response to the global coronavirus disease 2019 (COVID-19) pandemic has been focused on public safety, vaccine development, and treatment. A major challenge that emerged was the increase in vaccine hesitancy and medical mistrust. Social media exposure to vaccine-critical/antivaccination content has only furthered vaccine misinformation and disinformation and highlights the need for reputable, evidence-based vaccine information. Although vaccine hesitancy is not new, the number of preventable COVID-19 cases despite an effective and safe vaccine highlighted the need for healthcare providers to help patients make informed vaccination decisions. To achieve this goal, it is important to teach the next generation of healthcare providers approaches to discussing vaccine technology, vaccine side effects, and vaccination myths to combat vaccine hesitancy.

Few studies have described student perceptions of vaccines. One prepandemic study found that students had an overall positive attitude toward vaccines but a low perceived risk of infectious outbreaks and therefore a low vaccine prioritization.^1^ One study evaluating COVID-19 vaccine hesitancy among U.S. medical students found that most students had a positive attitude toward vaccines, but 23% were unwilling to get the COVID-19 vaccine themselves despite Food and Drug Administration approval.^2^ Students immediately willing to be vaccinated were more likely to trust public health experts, had fewer concerns about side effects, and agreed more with vaccine mandates. This highlights the need for a direct educational curriculum on vaccine development so that medical students will have more confidence about safety and effectiveness and can carry that forward in patient conversations. The only published studies on pandemic-era attitudes and education about vaccines assessed the impact of the pandemic on virtual learning.^3^^,^^4^ Thus, there is a knowledge gap in understanding current medical student attitudes to vaccines. More data is needed to guide medical students to become physicians who are knowledgeable about vaccines and can confidently guide patients through conversations about vaccine hesitancy.

Medical education in the U.S. is traditionally designed as a 4-year program, with a preclinical curriculum in the first 2 years comprising mostly didactic teachings followed by 2 years of clinical rotations in different hospital specialties. Current national medical education guidelines for accredited institutions, set forth by the Liaison Committee on Medical Education, require curricular content in the following areas: biomedical, behavioral, and social sciences; organ systems and differential diagnoses/treatments; clinical/translational research; problem-solving skills; healthcare disparities; medical ethics; communication skills; and interprofessionalism.^5^ How these content areas are presented to students, from duration to course design and what specific topics of medicine are emphasized, can vary widely across institutions. According to a 2022 American Association of Medical Colleges Curriculum Inventory survey, 125 of 140 accredited medical institutions include some topics related to vaccines and immunization in their curriculum; however, there remains no standardized formal vaccine education in most medical programs.^6^

This study was done at a large Pennsylvania medical school that, similar to most medical institutions, does not offer a formal vaccine curriculum in the preclinical or clinical years. We sought to discover current medical student attitudes on mandatory versus elective vaccine education. Furthermore, we questioned whether the pandemic stimulated medical student interest in vaccines, vaccine development, and skills to combat vaccine hesitancy as opposed to burnout from discussing vaccines with patients. Because curricular coverage of vaccines at this institution before the students’ entrance to hospital and start of clinical rotations is limited to their first-year microbiology course, we chose to assess preclinical students at the end of their first or second years of medical school (by which time they all have completed current vaccine-related coursework).

To complement the survey, we piloted a vaccine elective intervention at the same institution. The elective was the first focused education on the topic of vaccines and was developed by students with faculty supervision. We conducted pre- and postcourse assessments of enrolled students to assess the effectiveness of the education material provided during the course.

## METHODS

### Study Population and Measures

All studies of preclinical-year medical students were approved by the IRB and the university Research on Medical Students committee. Survey questions were newly designed by the authors and aimed to assess vaccine education topics, including policy, development, research, and communication, that are targetable through curricular changes.

A brief voluntary survey was administered to first- and second-year students at a single medical school in Pennsylvania using Qualtrics, a confidential and secure web-based platform. An anonymous survey link and study description were sent directly to the class e-mail lists for first- and second-year medical students. Formal discussion of vaccines is taught along with infectious diseases in the first-year microbiology course; thus, all participating students were expected to have received the school's current educational coverage of vaccines.

The survey was administered from May 1, 2022 to July 1, 2022 to align with completion of first- and second-year curricula. Students were given 2 months to respond. Three reminder emails were sent during this time to encourage participation. A total of 81 students (of an estimated 300 contacted) consented and completed the survey, giving a 27% response rate.

The survey consisted of 15 multiple-choice questions. Eleven were phrased as statements using a 5-point Likert-type scale to rate the degree of knowledge in vaccine-related topics, with 1 indicating *Hardly at all*, 2 indicating *To a small degree*, 3 indicating *To a moderate degree*, 4 indicating *To a considerable degree*, and 5 indicating *To a very high degree*. The other 4 questions were closed ended and prompted for responses of yes/no/maybe. The full survey questionnaire and answer options can be found in the Appendix Material (available online).

A voluntary precourse survey was sent to students enrolled in the Introduction to Vaccines elective 1 week before the course started using an anonymous Qualtrics e-mail survey. The survey consisted of the same 11 questions employing a 5-point Likert-type scale for rating knowledge of vaccine-related topics as described earlier. Of the 4 students initially enrolled, 3 completed the precourse survey. The medical school requires completion of postcourse surveys for all elective courses; with permission from the medical education directors, we added the same 11 scaled questions as an optional and voluntary section. Of the 3 students who attended all course sessions, 2 completed the postcourse survey.

A voluntary postcourse survey was sent to all faculty who taught in the Introduction to Vaccines elective course using an anonymous Qualtrics e-mail survey. The survey consisted of 1 open-ended and 4 multiple-choice questions. Of the 4 multiple-choice questions, 3 used yes/no responses to inquire about previous medical student teaching experience and opinions on vaccine education in formal and/or elective curricula. One used the 5-point Likert-type scale to assess the degree to which students currently received vaccine education in the curriculum. The open-ended question asked what improvements and/or additional topics faculty believed should be offered in the elective course.

### Statistical Analysis

All categorical data gathered in this study are reported as counts (number of responses) or frequency (percentage of total survey responses). Responses on the 5-point Likert-type scale are graphically depicted as 3 categories: very high (response of *very high degree* of knowledge), considerable (response of *considerable degree* of knowledge), or moderate to low (responses of *moderate degree, small degree*, or *hardly at all* and indicating insufficient knowledge). Median scores and IQRs between 1 and 5 on Likert-type scale responses for pre and postcourse surveys are reported to graphically depict trends in vaccine education topics.

Comparisons between groups were done using Kruskal–Wallis test given the categorical data set and smaller sample size. All tests are 2 tailed, and significance is set at alpha=0.05. All statistical analyses are done using IBM SPSS Statistics (Version 27) predictive analytics software.

## RESULTS

**Figure 1:**
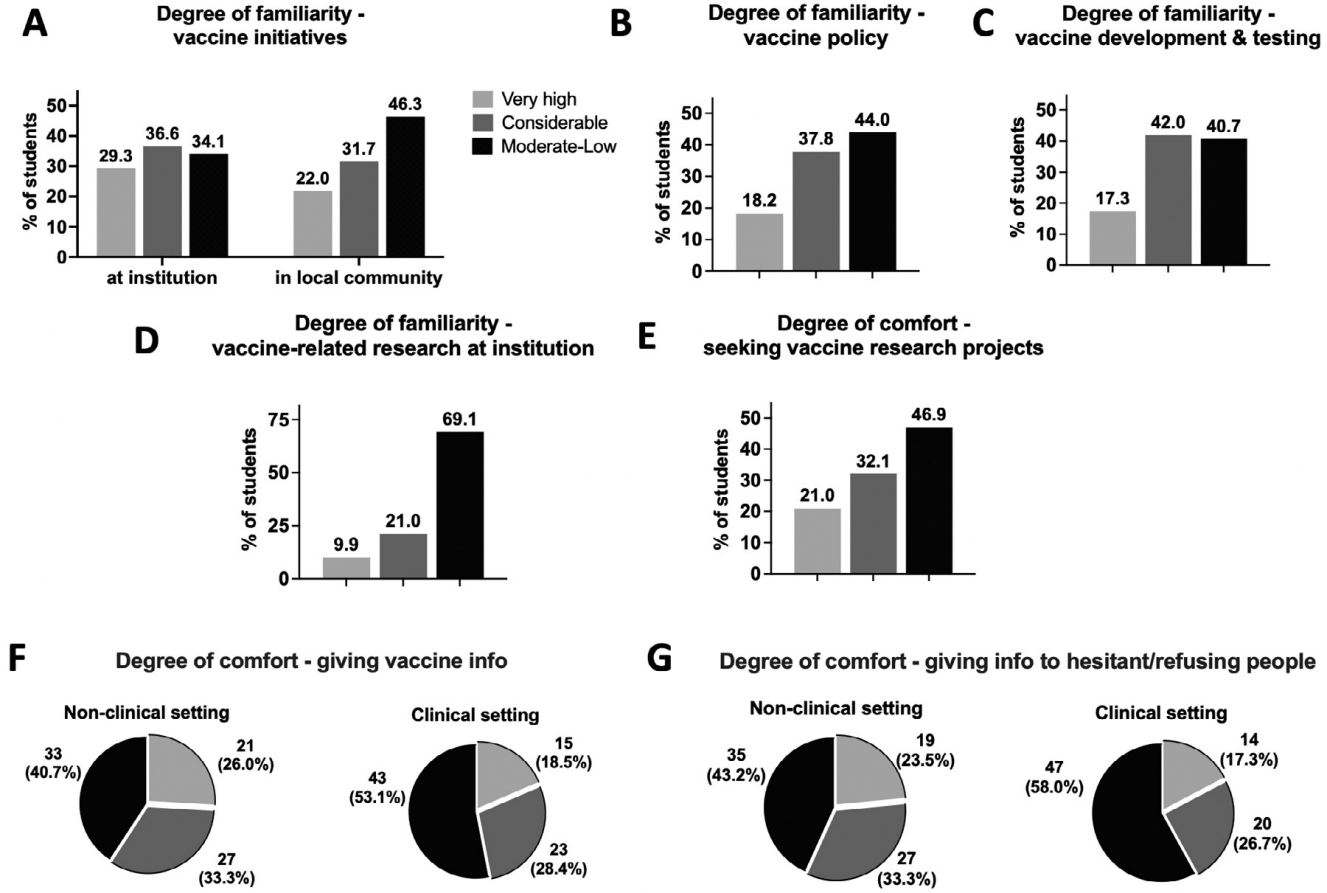
Perceived degree of awareness and/or comfort with several vaccine-related topics in first- and second-year medical students. Responses were stratified into 3 categories: very high degree of knowledge, considerable degree of knowledge, or insufficient (moderate-to-low) degree of knowledge. The frequencies of total participants are reported for **A–E**. The number and frequency of participants are reported for **F** and **G**.

A significant portion of participants reported insufficient (moderate-to-low) knowledge of vaccine initiatives at the medical school (34.1%) and within the local community (46.3%) (Figure 1A). Many students also reported insufficient knowledge of vaccine policy (44.0%) and vaccine development and testing (40.7%) (Figure 1B and C). Given the university's focus on medical student research and producing physician–scientists, we inquired about familiarity with vaccine-related research at the institution and how comfortable students felt seeking vaccine-related research projects. Most respondents (69.1%) reported insufficient knowledge of vaccine research at the university, and nearly half (46.9%) stated moderate-to-low comfort in seeking vaccine research projects (Figure 1D and E).

An important skill for future healthcare providers is comfort in discussing vaccines, particularly with vaccine-hesitant patients. Students at the medical school have weekly patient encounters starting in the first year; thus, all participants had some experience talking with patients in a clinical setting. In a nonclinical setting, many respondents stated moderate-to-low comfort in giving vaccine information overall (40.7%) and giving information to vaccine-hesitant people (43.2%) (Figure 1F and G). Most (>50%) participants reported lower comfort in discussing vaccines in a clinical setting: 53.1% felt moderate-to-low comfort in giving vaccine information, and 58.0% felt so in giving information to vaccine-hesitant people (Figure 1F and G).

**Figure 2:**
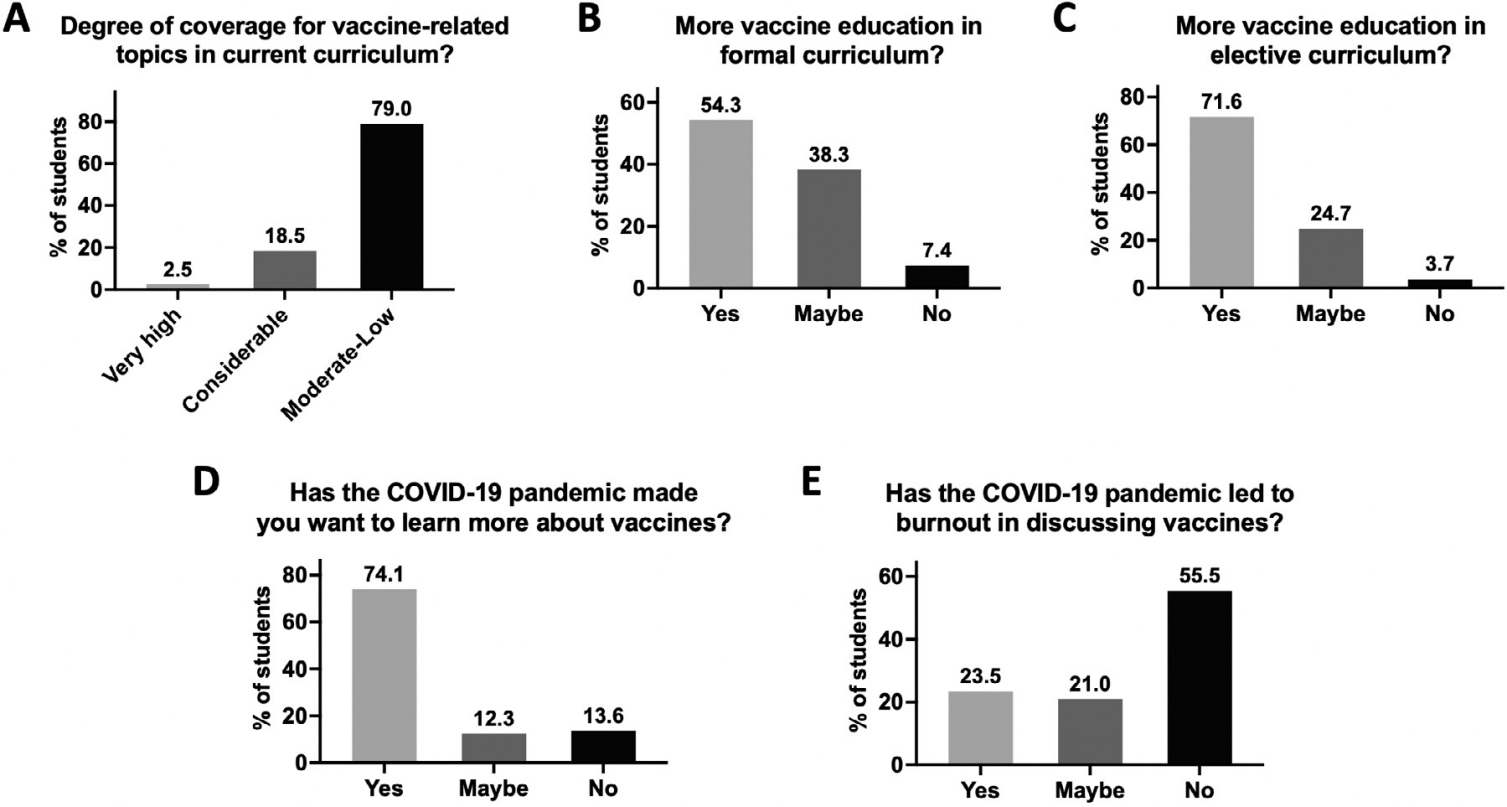
Student perceptions of curricular coverage of vaccines. (**A**) Perceived degree of coverage for vaccine-related topics in current curriculum. Responses were stratified by very high, considerable, or insufficient (moderate-to-low) degree. (**B, C**) Desire for vaccine education in formal and elective curricula. (**D, E**) Connections between willingness to learn about vaccines and the COVID-19 pandemic. Responses accepted for **B–E** are yes/no/maybe. The frequency of total participants is reported.

The students were asked to assess the degree of vaccine education in the current medical school curriculum; an overwhelming 79% of respondents reported insufficient coverage of vaccine-related topics (Figure 2A). Participants were also asked whether further vaccine education should be offered by formal and/or informal curricular changes. A total of 54.3% of students decisively wanted more vaccine education in the formal class curriculum (Figure 2B). The medical school offers electives, and 71.6% of students decisively wanted more vaccine education offered in the elective curriculum as well (Figure 2C).

Because these students were in the unique position of starting medical school during the COVID-19 pandemic, we wondered whether the pandemic had affected their willingness to learn about vaccines or possibly contributed to burnout in discussing vaccines. Reassuringly, 74.1% reported that the pandemic made them want to learn more about vaccines, and 55.5% said that this had not led to burnout from vaccine education (Figure 2D and E).

**Figure 3:**
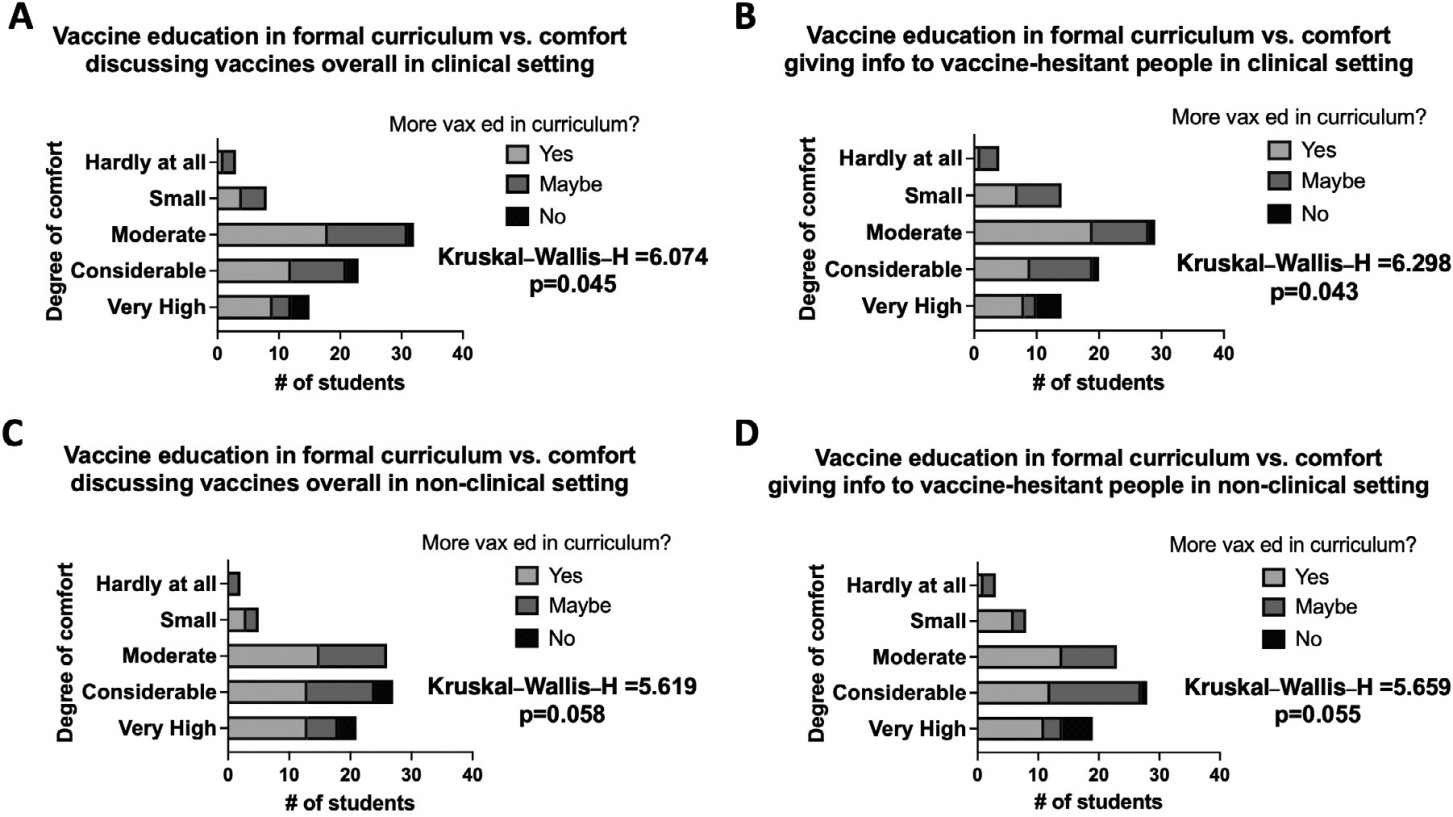
Associations between decisively wanting vaccine education in formal curriculum and comfort in discussing vaccines. Wanting formal curricular changes was associated with discomfort in giving information overall and to vaccine-hesitant people in (**A, B**) clinical settings. Wanting formal curricular changes also was associated with discomfort in giving vaccine information overall and to vaccine-hesitant people in (**C, D**) nonclinical settings. Values and significance for Kruskal–Wallis H-test are shown. Data are reported as total number of participants.

We sought to tailor curricular changes to student needs by assessing associations between current knowledge in vaccine-related topics and the desire for mandatory versus elective vaccine education (Appendix Table 1, available online). Choosing required/formal education was significantly associated with less comfort in discussing vaccines overall (*p*=0.045) and less comfort in discussing vaccines specifically with vaccine-hesitant/refusing people (*p*=0.043) in clinical settings (Figure 3A and B). Choosing formal education was also associated, albeit not significantly, with less comfort in discussing vaccines overall (*p*=0.058) and less comfort in discussing vaccines specifically with vaccine-hesitant/refusing people (*p*=0.055) in nonclinical settings (Figure 3C and D). Although choosing elective education did not associate significantly with knowledge of vaccine-related topics tested in this survey (Appendix Table 1, available online), electives remain a more accessible and targetable method of intervention.

Thus, to test the impact of targeted curricular changes, we designed a pilot elective teaching offered to first- and second-year medical students called Introduction to Vaccines. The course consisted of 4 sessions taught by expert faculty on community vaccine programs, vaccine research, policy and development of vaccines, and building comfort with discussing vaccines/navigating vaccine hesitancy. The class was capped at 10 students to foster open and honest discourse; 5 students enrolled, and 3 students completed the class requirements of attending all 4 sessions. Limited class sizes and the failure of many students to attend all 4 sessions highlight the difficulty of engaging students through extracurricular electives on top of already demanding course loads. Precourse and postcourse surveys of participants suggested that the elective teaching improved perceived knowledge and comfort, including comfort in discussing vaccines overall and with vaccine-hesitant people in both clinical and nonclinical settings (Appendix Table 2, available online).

We also surveyed course faculty, most of whom had previously taught vaccine-related topics to medical students, about their perceptions regarding current vaccine education at the school of medicine. A total of 80% (4 of 5) believed that medical students currently receive insufficient vaccine education in the formal curriculum, and all stated that more vaccine education should be offered in required and elective curricula (Appendix Table 3, available online). We asked faculty for suggestions on how elective vaccine education could be improved. Two recommended recruiting a larger elective class size, and 2 recommended an additional course session on the antivaccine industry and vaccine disinformation. One noted that electives are limited to interested students and suggested that more vaccine-related content in the required curriculum is needed.

## DISCUSSION

The objective of this study was to assess student knowledge of vaccine-related topics and attitudes toward vaccine education at a top U.S. medical school that, similar to most medical institutions, does not have a formal standardized vaccine curriculum. Our results suggest a perceived need among the surveyed medical students to improve upon vaccine education and gain comfort in discussing vaccines. Many medical students reported insufficient knowledge of vaccine policy, vaccine development, and vaccine-focused initiatives at their school and within the community, with 40%–60% also reporting discomfort in discussing vaccines in clinical and nonclinical settings. Importantly, the need for further vaccine education was well recognized, with 79% reporting insufficient coverage of vaccine topics in the current curriculum.

Insufficient coverage of vaccines in medical school curricula has been reported in other institutions and countries. A total of 70% of German medical students reported dissatisfaction with teaching on vaccine hesitancy and communication strategies for vaccine information.^7^ A U.S. study found that only 40% of medical students expressed knowledge of the human papillomavirus vaccine, and 40% felt comfortable counseling patients.^8^ A survey of French medical students noted that one third felt unprepared to communicate with patients about vaccines or navigate vaccine hesitancy.^9^ Attending medical school during the COVID-19 pandemic may have increased students’ awareness of vaccine education deficits; a survey of German medical students showed that participants were more likely to view vaccinations for a broader range of diseases as important and more likely to agree with compulsory vaccination during than before the pandemic.^10^

We found that many students wanted more formal and elective vaccine education, and most participants reported that the COVID-19 pandemic increased their interest in vaccine education. As future healthcare providers, comprehensive education about vaccines is important for medical students. Improved vaccine knowledge on the part of medical professionals leads to greater confidence and perception of vaccines by healthcare professionals and influences their recommendations to patients.^11^^,^^12^ Importantly, vaccine hesitancy is prevalent among medical students themselves.^13^ A survey of medical students revealed that 23% were unwilling to get the COVID-19 vaccine immediately upon Food and Drug Administration approval.^2^ Medical professionals are a trusted source of vaccine information and have an important role in modeling behaviors to increase vaccination.^14^

Both formal and elective curricular changes have had measured success in medical student vaccine education. A formal curriculum addendum improved knowledge of human papillomavirus vaccines and student comfort in talking to vaccine-hesitant parents, and elective teaching at an Italian medical institution augmented knowledge of vaccine topics.^15^^,^^16^ We sought to identify student needs by associating degree of knowledge in vaccine-related topics with wanting mandatory versus elective vaccine education. Interestingly, wanting mandatory vaccine education was associated with discomfort in discussing vaccines both overall and with vaccine-hesitant people in clinical settings. Findings suggest adding formal curricular changes that center on addressing vaccine hesitancy and improving communication strategies for vaccine information.

We piloted an elective course that taught first- and second-year medical students about vaccine-related topics using lectures, open discussion, speaker panels, and case-based role playing. Postcourse surveys showed improvement in perceived knowledge and comfort in all vaccine-related topics we assessed, including comfort in discussing vaccines overall and with vaccine-hesitant people in both clinical and nonclinical settings. Similar interventions combining lectures and practice-based learning have improved vaccine knowledge when piloted in domestic and French medical schools and have shown promise for both preclinical trainees and postgraduate healthcare providers.^9^^,^^17^^,^^18^

It is important to note the limited sample size for this survey and subsequent pilot elective. Student participation in medical and health professional education research is frequently low and requires improvement, with reported disincentives including time commitment, survey fatigue, an unclear understanding of the value of education research, and ethical considerations of educators/instructors as researchers.^19^ One well-studied way to make students feel valuable and ultimately improve engagement in education research is by directly involving them in curricular design and changes. The study of a student curricular board that trained students in medical program evaluation and placed them on administrative committees at the University of Illinois College of Medicine in Chicago showed that involved students were more likely to be aware of specific program initiatives and expressed increased interest in academic medicine.^20^ A similar program at Baylor College of Medicine where students worked alongside faculty to cocreate a new medical curriculum was favorably received by students and fostered professional and interpersonal growth for student participants.^21^

In addition, several of the measured variables, including community-based initiatives, research, and extracurricular learning, are lacking in medical student participation and engagement. One meta-analysis showed the proportion of medical students reporting interest in research is significantly higher than that of students involved in research projects, and about half of students not involved with research cited being deterred by financial factors—both in short-term financial burden and financial concerns of a research career.^22^ Students at a medical institution in Brazil also reported the limited number of available positions, lack of institutional support, poor time management, risk of lowered academic performance, and lack of physical and financial resources as disincentives for participating in community-based extracurricular activities.^23^ The American Association of Medical Colleges identified that only one third of graduating medical students report field experience in service,^24^ and many medical schools do not incorporate volunteering into their mission statement or program requirements.^25^ Yet, participation in longitudinal volunteer programs is shown to improve communication, strengthen interpersonal and teaching skills, and help students identify community needs.^25^

Through this study, we were able to identify a perceived gap in vaccine education among medical students, identify vaccine-related topic areas where students felt less knowledgeable, and pilot a successful educational intervention on the basis of these findings that markedly improved student comfort with discussing vaccines.

### Limitations

Limitations of our study included a small sample size of 81 participants. On the basis of average class sizes per year, we estimate we contacted ∼300 students and had a 27% survey response rate. Another limitation is the study of a single medical institution. In addition, although an elective intervention was tested, a formal intervention could not be assessed given the difficulty in developing mandatory curricular changes. Although we could measure the outcomes of elective education, this was a pilot intervention that was limited to a small number of students. We aim to improve this elective on the basis of identified student needs and the suggestions of participants and faculty as well as expand class size for future courses.

## CONCLUSIONS

In conclusion, we uncovered a need among medical students to improve vaccine teaching, with most participants reporting insufficient coverage of vaccine topics in their curriculum. Notably, the desire to learn more about vaccines has been strengthened by experiencing the COVID-19 pandemic. We also identified associations between discomfort with vaccine-related topics and wanting mandatory versus elective vaccine education, which allows us to tailor curricular changes to student needs. On the basis of our findings, vaccine curricula should be tailored to the educational needs of students, taking into consideration the benefits and disadvantages of mandatory formal versus optional elective classes.

## CRediT authorship contribution statement

**Jorna Sojati:** Conceptualization, Data curation, Formal analysis, Methodology, Software, Writing – original draft. **Anjana Murali:** Data curation, Writing – original draft. **Glenn Rapsinski:** Supervision, Writing – review & editing. **John V. Williams:** Supervision, Writing – review & editing.

## Appendix Supplementary materials

Image, application 1

## Footnotes

Supplementary material associated with this article can be found, in the online version, at doi:10.1016/j.focus.2023.100178.
