# Supplementary material for: Do Not Throw Away Your Shot: Pilot Study in Improving Medical School Curricula Through Focused Vaccine Education
Source: AJPM Focus. 2023 Dec 23;3(2):100178. doi: 10.1016/j.focus.2023.100178 (PMC10835123; doi:10.1016/j.focus.2023.100178)
Supplement: Supplementary file 1 [file mmc1.docx]

**Appendix Material**

**Table 1.** Associations between wanting formal (top) or elective (bottom) vaccine education (responses of: yes, maybe, or no) and all assessed vaccine-related topics (uses 5-point Likert Scale: 1=Hardly at all; 2=Small degree; 3=Moderate degree; 4=Considerable degree; 5=Very high degree). Significance for Kruskal-Wallis H-test is reported for tested variables. Parentheses are used to denote survey question (Q) corresponding to each measured variable in Appendix.

***Formal* vaccine education (vs.)**

| **Cross-tabulating variable** | **Median (IQR)** | **p-value** |
| --- | --- | --- |
| Initiatives at institution (**Q1**)  Yes  Maybe  No | 4.00 (2.00)  4.00 (1.00)  5.00 (1.00) | 0.085 |
| Initiatives in local community (**Q2**)  Yes  Maybe  No | 4.00 (1.00)  4.00 (1.00)  4.00 (1.50) | 0.948 |
| Policy (**Q3**)  Yes  Maybe  No | 4.00 (1.00)  4.00 (1.00)  3.50 (2.00) | 0.646 |
| Development and testing (**Q4**)  Yes  Maybe  No | 4.00 (1.00)  4.00 (1.00)  4.50 (1.50) | 0.283 |
| Research at institution (**Q5**)  Yes  Maybe  No | 3.00 (2.00)  3.00 (2.00)  3.50 (2.50) | 0.776 |
| Seeking research projects (**Q6**)  Yes  Maybe  No | 4.00 (1.75)  3.00 (2.00)  4.00 (2.25) | 0.559 |
| Discussing overall – non-clinical setting (**Q7**)  Yes  Maybe  No | 4.00 (2.00)  4.00 (1.00)  4.50 (1.00) | 0.058 |
| Discussing with vaccine-hesitant people– non-clinical setting (**Q8**)  Yes  Maybe  No | 4.00 (1.75)  4.00 (1.00)  5.00 (1.00) | 0.055 |
| Discussing overall –clinical setting (**Q9**)  Yes  Maybe  No | 3.00 (1.00)  3.00 (1.00)  4.50 (1.25) | ***0.045*** |
| Discussing with vaccine-hesitant people–  clinical setting (**Q10**)  Yes  Maybe  No | 3.00 (1.00)  3.00 (2.00)  5.00 (1.50) | ***0.043*** |

***Elective* vaccine education (vs.)**

| **Cross-tabulating variable** | **Median (IQR)** | **p-value** |
| --- | --- | --- |
| Initiatives at institution (**Q1**)  Yes  Maybe  No | 4.00 (2.00)  4.00 (1.00)  5.00 (1.00) | 0.789 |
| Initiatives in local community (**Q2**)  Yes  Maybe  No | 4.00 (1.25)  4.00 (1.75)  3.00 (1.50) | 0.249 |
| Policy (**Q3**)  Yes  Maybe  No | 4.00 (1.00)  4.00 (1.00)  3.00 (2.00) | 0.265 |
| Development and testing (**Q4**)  Yes  Maybe  No | 4.00 (1.00)  3.50 (1.00)  4.00 (1.00) | 0.622 |
| Research at institution (**Q5**)  Yes  Maybe  No | 3.00 (2.00)  2.00 (2.00)  3.00 (2.00) | 0.658 |
| Seeking research projects (**Q6**)  Yes  Maybe  No | 4.00 (2.00)  3.00 (2.00)  3.00 (1.00) | 0.616 |
| Discussing overall – non-clinical setting (**Q7**)  Yes  Maybe  No | 4.00 (2.00)  4.00 (1.00)  4.00 (2.00) | 0.780 |
| Discussing with vaccine-hesitant people– non-clinical setting (**Q8**)  Yes  Maybe  No | 4.00 (1.25)  4.00 (1.00)  4.00 (1.00) | 0.488 |
| Discussing overall –clinical setting (**Q9**)  Yes  Maybe  No | 3.00 (1.00)  4.00 (2.50)  4.00 (2.00) | 0.953 |
| Discussing with vaccine-hesitant people–  clinical setting (**Q10**)  Yes  Maybe  No | 3.00 (1.00)  3.00 (2.75)  4.00 (0.00) | 0.446 |

**Table 2.** Median scores and range of perceived awareness and/or comfort in several vaccine-related topics in pre-course (left) vs. post-course (right) assessments of first- and second-year medical students participating in the pilot Intro to Vaccines elective course (uses 5-point Likert Scale: 1=Hardly at all; 2=Small degree; 3=Moderate degree; 4=Considerable degree; 5=Very high degree).

Student Survey Responses:

| **Student survey question** | **Pre-Course Responses**  **(n=3 participants)** | **Post-Course Responses**  **(n=2 participants)** |
| --- | --- | --- |
| Degree of Awareness of Vaccine Initiatives at Institution | 2/3 rate Moderate;  1/3 rate Considerable  **Median 3; Range (3-4)** | 1/2 rate Considerable; 1/2 rate Very high  **Median 4.5; Range (4-5)** |
| Degree of Awareness of Initiatives in Local Community | 1/3 rate Hardly at all; 1/3 rate Small; 1/3 rate Moderate  **Median 2; Range (1-3)** | 1/2 rate Considerable; 1/2 rate Very high  **Median 4.5; Range (4-5)** |
| Degree of Awareness of Vaccine Policy | 1/3 rate Hardly at all; 2/3 rate Moderate  **Median 3; Range (1-3)** | 1/2 rate Considerable; 1/2 rate Very high  **Median 4.5; Range (4-5)** |
| Degree of Awareness of Vaccine Development & testing | 1/3 rate Small; 2/3 rate Moderate  **Median 3; Range (2-3)** | 2/2 rate Very high  **Median 5; Range (5-5)** |
| Degree of Awareness of Vaccine Research at Institution | 3/3 rate Moderate  **Median 3; Range (3-3)** | 1/2 rate Considerable; 1/2 rate Very high  **Median 4.5; Range (4-5)** |
| Degree of Comfort Seeking Vaccine Research Projects | 3/3 rate Considerable  **Median 4; Range (4-4)** | 1/2 rate Considerable; 1/2 rate Very high  **Median 4.5; Range (4-5)** |
| Degree of Comfort Discussing Vaccines - Non-Clinical Setting | 2/3 rate Moderate;  1/3 rate Considerable  **Median 3; Range (3-4)** | 1/2 rate Considerable; 1/2 rate Very high  **Median 4.5; Range (4-5)** |
| Degree of Comfort Discussing with Vaccine-Hesitant People – Non-Clinical Setting | 2/3 rate Small;  1/3 rate Considerable  **Median 2; Range (2-4)** | 1/2 rate Considerable; 1/2 rate Very high  **Median 4.5; Range (4-5)** |
| Degree of Comfort Discussing Vaccines - Clinical Setting | 1/3 rate Moderate;  2/3 rate Considerable  **Median 4; Range (3-4)** | 1/2 rate Considerable; 1/2 rate Very high  **Median 4.5; Range (4-5)** |
| Degree of Comfort Discussing with Vaccine-Hesitant People –Clinical Setting | 1/3 rate Hardly at all;  2/3 rate Moderate  **Median 3; Range (1-3)** | 1/2 rate Considerable; 1/2 rate Very high  **Median 4.5; Range (4-5)** |

**Table 3.** Results of survey to faculty members who taught the pilot Introduction to Vaccines elective course to first- and second-year medical students.

| **Faculty survey question** | **Responses (n=5 participants)** |
| --- | --- |
| Have you worked with or taught students on vaccine-related topics in the past? | **3/5 (60%) Yes**, 2/5 No |
| To what degree do you think students receive vaccine education in formal curriculum? | 1/5 Very high degree,  **4/5 (80%) Moderate-low degree** |
| Do you think students should have vaccine education offered in formal curriculum? | **5/5 (100%) Yes**, 0/5 No |
| Do you think students should have vaccine education offered in elective curriculum? | **5/5 (100%) Yes**, 0/5 No |

**Questionnaire: Medical Student Vaccine Education Perceptions Survey**

Statement A. I understand and acknowledge that this survey will not use any personal identifiers and that my answers will remain completely anonymous.

· Yes

· No

Statement B. I consent to my answers for this survey being used for medical education research and quality improvement purposes.

· Yes

· No

1. I am familiar with COVID-19 and other **vaccine initiatives** at [institution name]

· To a very high degree

· To a considerable degree

· To a moderate degree

· To a small degree

· Hardly at all

2. I am familiar with COVID-19 and other **vaccine initiatives** in the local community

· To a very high degree

· To a considerable degree

· To a moderate degree

· To a small degree

· Hardly at all

3. I am familiar with national-level, state, and/or local-level **vaccine policy**

· To a very high degree

· To a considerable degree

· To a moderate degree

· To a small degree

· Hardly at all

4. I am familiar with how vaccines are **developed & tested**

· To a very high degree

· To a considerable degree

· To a moderate degree

· To a small degree

· Hardly at all

5. I am aware of **vaccine-related research** at [institution name]

· To a very high degree

· To a considerable degree

· To a moderate degree

· To a small degree

· Hardly at all

6. I feel comfortable seeking out **vaccine-related research projects**

· To a very high degree

· To a considerable degree

· To a moderate degree

· To a small degree

· Hardly at all

7. I feel comfortable giving vaccine information in a **non-clinical** setting

· To a very high degree

· To a considerable degree

· To a moderate degree

· To a small degree

· Hardly at all

8. I feel comfortable discussing vaccines with vaccine-hesitant/refusing people in a **non-clinical setting**

· To a very high degree

· To a considerable degree

· To a moderate degree

· To a small degree

· Hardly at all

9. I feel comfortable giving vaccine information in a **clinical setting**

· To a very high degree

· To a considerable degree

· To a moderate degree

· To a small degree

· Hardly at all

10. I feel comfortable discussing vaccines with vaccine-hesitant/refusing people in a **clinical** setting

· To a very high degree

· To a considerable degree

· To a moderate degree

· To a small degree

· Hardly at all

11. To what degree has the medical school curriculum (so far) covered vaccine-related topics?

· To a very high degree

· To a considerable degree

· To a moderate degree

· To a small degree

· Hardly at all

12. Do you think [institution name] students should have more vaccine education offered in the formal curriculum?

· Yes

· Maybe

· No

13. Do you think [institution name] students should have more vaccine education offered as an elective curriculum?

· Yes

· Maybe

· No

14. Has the COVID-19 pandemic made you want to learn more about vaccines, vaccine development, skills to combat vaccine hesitancy?

· Yes

· Maybe

· No

15. Has the COVID-19 pandemic made you burned out from discussing vaccines with patients?

· Yes

· Maybe

· No
